# Eveningness is associated with sedentary behavior and increased 10-year risk of cardiovascular disease: the SCAPIS pilot cohort

**DOI:** 10.1038/s41598-022-12267-5

**Published:** 2022-05-17

**Authors:** Mio Kobayashi Frisk, Jan Hedner, Ludger Grote, Örjan Ekblom, Daniel Arvidsson, Göran Bergström, Mats Börjesson, Ding Zou

**Affiliations:** 1 Center for Sleep and Vigilance Disorders, Department of Internal Medicine and Clinical Nutrition, Institute of Medicine, Sahlgrenska Academy, University of Gothenburg, Medicinaregatan 8b, Box 421, 40530 Gothenburg, Sweden; 2 Sleep Disorders Center, Department of Pulmonary Medicine, Sahlgrenska University Hospital, Gothenburg, Sweden; 3 Department of Physical Activity and Health, The Swedish School of Sport and Health Sciences, Stockholm, Sweden; 4 Center for Health and Performance, Institute of Food, Nutrition and Sports Science, Gothenburg University, Gothenburg, Sweden; 5 Department of Molecular and Clinical Medicine, Institute of Medicine, Sahlgrenska Academy, Gothenburg University, Gothenburg, Sweden; 6 Department of Clinical Physiology, Region Västra Götaland, Sahlgrenska University Hospital, Gothenburg, Sweden; 7 Sahlgrenska University Hospital/Östra, Gothenburg, Sweden

**Keywords:** Cardiology, Risk factors

## Abstract

Chronotype reflects individual preferences for timing activities throughout the day, determined by the circadian system, environment and behavior. The relationship between chronotype, physical activity, and cardiovascular health has not been established. We studied the association between chronotype, physical activity patterns, and an estimated 10-year risk of first-onset cardiovascular disease (CVD) in the Swedish CArdioPulmonary bioImage Study (SCAPIS) pilot cohort. A cross-sectional analysis was performed in a middle-aged population (n = 812, 48% male). Self-assessed chronotype was classified as extreme morning, moderate morning, intermediate, moderate evening, or extreme evening. Time spent sedentary (SED) and in moderate to vigorous physical activity (MVPA) were derived from hip accelerometer. The newly introduced Systematic COronary Risk Evaluation 2 (SCORE2) model was used to estimate CVD risk based on gender, age, smoking status, systolic blood pressure, and non-HDL cholesterol. Extreme evening chronotypes exhibited the most sedentary lifestyle and least MVPA (55.3 ± 10.2 and 5.3 ± 2.9% of wear-time, respectively), with a dose-dependent relationship between chronotype and SED/MVPA (p < 0.001 and p = 0.001, respectively). In a multivariate generalized linear regression model, extreme evening chronotype was associated with increased SCORE2 risk compared to extreme morning type independent of confounders (β = 0.45, SE = 0.21, p = 0.031). Mediation analysis indicated SED was a significant mediator of the relationship between chronotype and SCORE2. Evening chronotype is associated with unhealthier physical activity patterns and poorer cardiovascular health compared to morning chronotype. Chronotype should be considered in lifestyle counseling and primary prevention programs as a potential modifiable risk factor.

## Introduction

Circadian chronotype refers to an individual’s propensity to time sleep and various other activities throughout the 24-h day. Evening chronotypes, or “night owls” tend to stay up late and to have more energy later in the day, whereas morning chronotypes, or “morning larks” tend to get up early and are more energetic earlier in the day. Chronotype is closely correlated to the endogenous circadian timing system determined by molecular clocks, but also encompasses behavioral and environmental factors. Factors such as light, ambient temperature, food intake, exercise, comorbidities, age, and social constraints can influence chronotype^1–3^.

Low levels of physical activity, a well-established risk factor for cardiovascular and metabolic disease, is associated with increased all-cause mortality^4–9^. For example, Ekelund and colleagues found that sedentary time of 9.5 h or more per day was associated to a significantly higher risk of death, although other studies have found other cut-offs^5–9^. Many population-based studies using self-reported physical activity data suggest that evening chronotype is associated with lack of physical activity^10–13^. Wennman et al.^11^ found that none or very low physical activity is more than twice as likely in evening types compared to morning chronotypes, and morning tiredness among evening types is an important factor in this relationship. There are few studies utilizing objective physical activity assessment to assess the impact of chronotype. Hisler et al.^14^ studied frequency and timing of physical activity using data from Fitbit Zips in relation to diurnal preference in healthy university students and faculty members in the United States. They found that diurnal preference predicted exercise frequency, and that exercise was more likely to occur if performed in congruence with the individual’s diurnal preference. Nauha and colleagues recently studied physical activity and sedentary time in relation to chronotype using wrist-worn accelerometers in a Finish middle-aged population. They found that evening chronotype was associated to low physical activity, and among men, high sedentary time, whereas there was no association between chronotype and sedentary time among women^15^. Which time of the day was associated with physical inactivity in evening chronotype subjects was not reported.

Physiological parameters of the cardiovascular system are also affected by the circadian system and exhibit diurnal variations. For instance, blood pressure fluctuates throughout the day, dipping in the night and reaching a peak during the mid-morning^16^. Abnormal evening blood pressure dipping patterns are prognostic markers of cardiovascular disease (CVD)^17^. The link between diurnal preference, physical activity and CVD has not been fully established. Physical activity can act as a zeitgeber, influencing the circadian system^2^. There is also evidence suggesting that the metabolic effects of physical activity are time-dependent, where physical activity in the active phase (during the day in humans) favors carbohydrate metabolism^18,19^. Whether this translates to improved CV health is still unclear. Evening chronotype is a diurnal preference reflecting potential circadian disruption and misalignment and has been associated to increased morbidities and mortality^20^. In the current study, we aimed to investigate the relationship between chronotype, objectively measured physical activity patterns, and 10-year first-onset CVD risk assessed by the Systematic COronary Risk Evaluation 2 (SCORE2)^21^. We hypothesized that evening chronotype is associated with a sedentary lifestyle as well as increased CVD risk in a middle-aged population. We further speculate that physical activity partially mediates the relationship between chronotype and CVD risk.

## Methods

### Study population

The Swedish CArdioPulmonary BioImage Study (SCAPIS) was initiated with the objective of better understanding CVD, chronic obstructive pulmonary disease, and associated metabolic disease, and has previously been described in detail^22^. As a pilot cohort to assess the feasibility of the SCAPIS design and to estimate the frequency of pathological findings amongst participants, 2243 individuals aged between 50–64 years were randomly invited from two known residential areas of high and low socioeconomic status (SES) in Gothenburg, Sweden in 2012^23^. The exclusion criteria was the inability to understand spoken and written Swedish for purposes of informed consent. 1111 individuals agreed to participate and were included in the study^22,24^. Participants underwent extensive examination with imaging and functional testing, blood sampling, objective assessment of physical activity, anthropometric measurements, and questionnaires assessing health and lifestyle. SCAPIS has been approved as a multicenter study by the ethics committee at Umeå University (Dnr 2010–228-31 M). The current cross-sectional analysis was approved by the Regional Ethical Review Board in Gothenburg (Dnr 638–16). Written informed consent was obtained from all study participants. All methods were performed in accordance with the relevant guidelines and regulations.

The data collection was performed at the SCAPIS center, Sahlgrenska University Hospital, Gothenburg, Sweden and data analysis was performed at the Center for Sleep and Vigilance Disorders, University of Gothenburg, Sweden.

### Chronotype and sleep assessment

Chronotype was obtained by means of a multiple-choice question: “Try to specify to what extent you consider yourself a morning or evening type.” The possible answers were (1) I am a distinct morning person (extreme morning type); (2) I am a morning person to a certain degree (moderate morning type); (3) I am neither a morning nor evening person (intermediate type); (4) I am an evening person to a certain degree (moderate evening type); (5) I am a distinct evening person (extreme evening type); (6) I do not know. Participants who did not answer or indicated “I do not know” were excluded from the analysis. Leisure bedtime and wake-up time information was derived from the question, “On a day off from work (for example when you are on vacation), what time do you go to bed, and what time do you get up?”. Mid-sleep time was calculated as the mid-point of bedtime and wake-up time (available in 685 subjects)^25^. Subjective sleep quality was assessed by Pittsburg Sleep Quality Index (PSQI) questionnaire, which assesses subjective sleep quality, sleep latency, sleep duration, habitual sleep efficiency, sleep disturbances, use of sleep medications, and daytime dysfunction over a one-month period^26^. Scores range from 0–21 points, and participants with PSQI score > 5 were considered to have poor sleep quality. Habitual sleep duration was derived from a multiple-choice question with seven options: (1) 4 h or less; (2) 5 h; (3) 6 h; (4) 7 h; (5) 8 h; (6) 9 h; (7) 10 h or more.

### Physical activity assessment

Physical activity was measured using a triaxial accelerometer with sampling frequency of 30 Hz (ActiGraph GT3X and GT3X + , ActiGraph, LCC, Pensacola, FL, USA). Participants were instructed to wear the accelerometer on the right hip for seven consecutive days during all waking hours, except during water-based activities. Those with at least 600 min per day of wear time for at least 4 days were included in the analysis. The distribution of valid days was 69.7% for 7 days, 18.2% for 6 days, 8.6% for 5 days, and 3.4% for 4 days. Data was downloaded using the ActiLife software (v. 6.10.1, ActiGrah LCC, Pensacola, FL, USA). Vector magnitude counts per minute (cpm) were used to classify time spent sedentary (SED: 0–199 cpm), in light intensity physical activity (LIPA: > 199 & < 2690 cpm), and in moderate to vigorous intensity physical activity (MVPA: ≥ 2690 cpm)^27^. These cut-points have been used in previous SCAPIS studies and have shown high prediction accuracy^27–29^.

Average daily percentage of SED and MVPA over the measurement period was used in the analysis. Total volume of physical activity was expressed as mean cpm of wear time. A bout of SED was defined as a period of 20 consecutive minutes or more below 199 cpm with no allowance for interruption above the threshold. A bout of MVPA was defined as a period of 10 consecutive minutes above and equal to 2690 cpm, with an allowance of up to 2 minutes below this threshold, in accordance with Swedish public health guidelines^30^. Percentages of SED and MVPA in the morning (06:00 to 12:00), afternoon (12:00 to 18:00) and evening (18:00 to 00:00) were further calculated. LIPA was not included in the analysis since it was strongly negatively correlated to SED (r = − 0.949).

### Estimation of the 10-year risk of first-onset CVD

Traditional risk factors of CVD are frequently grouped together to improve risk prediction instead of relying on single risk factors. SCORE2 is the latest version of the European risk scoring model which estimates the 10-year risk of first-onset CVD based on gender, age, smoking status, systolic blood pressure (SBP), and non-high density lipoprotein (non-HDL) cholesterol^21^. Based on country-specific CVD mortality, European countries were divided into four groups (low, moderate, high, and very high risk) of which Sweden was classified as a moderate risk country. The SCORE2 risk chart for Sweden was used to estimate risk for each participant. In the current study, smoking status was derived from a self-report (non-smoker, occasional smoker, former smoker and current active smoker). SBP was measured in the supine position twice in each arm with an automatic device (Omron M10-IT. Omron Health Care Co., Kyoto, Japan) and calculated as the average of the assessments registered in the arm with the highest mean SBP^31^. Cholesterol was analyzed from a fasting venous blood sample obtained at the department of clinical chemistry, Sahlgrenska University Hospital. Non-HDL was calculated as total cholesterol minus HDL cholesterol. The calculated SCORE2 was reported as a percentage.

### Confounding variables

Information on education, work status, lifestyle, and comorbidities were collected from questionnaire data. University education (yes/no) was used to describe the participants’ education level. Income-related work (yes/no) was used to determine work status. Alcohol consumption was assessed using the Alcohol Use Disorders Identification Test (AUDIT) where a total score of 5 or higher in men and 4 or higher in women was classified as unhealthy drinking^32^. Information about depression symptoms (yes/no) was based on the question: “During the past 12 months, have you experienced a period of two weeks or more of feeling sad, downhearted or depressed?”. Hypertension (yes/no) was defined as at least one of the following criteria: self-reported treatment of hypertension, SBP ≥ 140 mmHg, or diastolic blood pressure ≥ 90 mmHg.

### Statistics

Statistical analyses were performed using SPSS 27.0 (IBM, Armonk, NY, USA). Data are shown as percentage, mean ± standard deviation (SD) or 95% confidence interval (CI). Chi-square tests and analysis of variance (ANOVA) tests with Bonferroni post-hoc tests were applied for categorical and continuous variables respectively for between-groups comparisons. The associations between chronotype, physical activity and cardiovascular risk were analyzed using generalized linear regression models, adjusting for age, gender, body mass index (BMI), waist circumference, SES, university education, work status, smoking, unhealthy drinking, depression symptoms and comorbidities. Mediation analyses were conducted using the PROCESS procedure for SPSS version 3.5.3 to determine whether there was a mediator relationship between chronotype (predictor), physical activity patterns (mediator), and CVD risk (outcome). Bootstrapping methods with 5000 samples were used to obtain confidence limits. Comparison between chronotype and mid-sleep time on non-working-days were performed in a subgroup of participants. A p-value less than 0.05 (two-tailed) was considered statistically significant.

## Results

**Figure 1:**
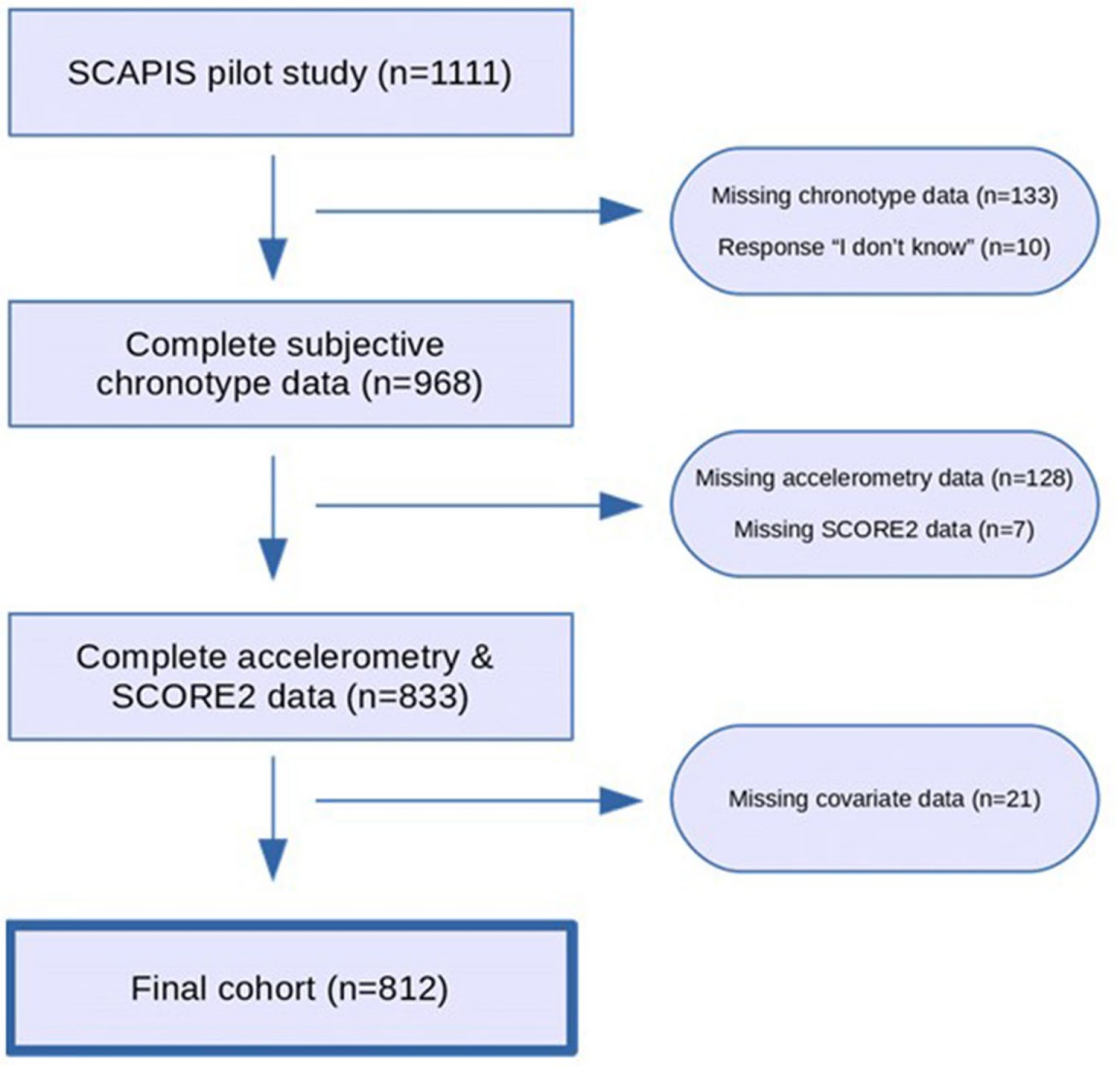
Study flowchart.

A total of 812 subjects (48% male, age 57.6 ± 4.4 years) were included in the final analysis (Fig. 1). Compared to excluded subjects in the SCAPIS pilot cohort, subjects in the current analysis were generally in better health (Table S1).

### Characteristics by chronotype

The distribution of chronotype was 18.7% extreme morning; 20.9% moderate morning; 25.0% intermediate; 18.8% moderate evening; 16.5% extreme evening. Morning types were predominantly female. Body composition, as measured by BMI as well as waist circumference, was generally healthier in extreme morning types and unhealthier in extreme evening types (p = 0.009 and 0.001, respectively). Depression symptoms were significantly different across the chronotypes. They were most common among extreme evening types, followed by extreme morning types. Intermediate types had the lowest frequency of depressive symptoms (p = 0.005). Current working status varied significantly across the chronotypes, with the least currently employed participants in the extreme evening group compared to more employed participants in especially the extreme morning and moderate evening groups. The characteristics of the participants across the chronotypes are summarized in Table 1.

**Table 1:** Characteristics and physical activity patterns of the population.

	Total (n = 812)	Extreme morning (n = 152)	Moderate morning (n = 170)	Intermediate (n = 203)	Moderate evening (n = 153)	Extreme evening (n = 134)	p-value
Male (n [%])	393 (48.4)	56 (36.8)	74 (43.5)	109 (53.7)	86 (56.2)	68 (50.7)	**0.003**
Age (years)	57.6 (57.3–57.9)	57.2 (56.5–57.9)	58.1 (57.4–58.7)	57.7 (57.1–58.2)	57.7 (56.9–58.4)	57.6 (56.8–58.4)	0.55
Body mass index (kg/m^2^)	27.0 (26.7–27.3)	26.3 (25.7–27.0)	27.2 (26.5–27.9)	27.2 (26.6–27.8)	26.5 (25.9–27.1)	28.0 (27.2–28.7)	**0.009**
Waist circumference (cm)	94.6 (93.7–95.4)	91.4 (89.6–93.3)	94.4 (92.5–96.3)	95.7 (93.9–97.6)	94.2 (92.5–95.9)	97.0 (95.0–99.1)	**0.001**
Low socioeconomic status (%)	45.1	48.0	37.6	46.8	42.5	51.5	0.13
University education (%)	39.5	36.8	39.4	37.4	43.8	41.0	0.71
Income-related job (%)	78.9	82.2	78.2	77.8	85.0	70.9	**0.045**
Current/occasional/former smoker (%)	55.4	47.4	55.3	55.7	58.8	60.4	0.19
Unhealthy alcohol consumption (%)	28.0	22.4	24.1	33.5	28.8	29.9	0.14
Self-reported sleep duration ≤ 6 h (%)	35.8	39.3	38.2	34.5	28.1	39.6	0.19
PSQI score > 5 (%, n = 717)	47.3	50.4	48.7	42.5	47.4	49.2	0.65
Depression symptoms (%)	25.6	28.3	21.8	20.2	24.2	37.3	**0.005**
Hypertension (%)	31.4	26.3	28.2	31.5	34.0	38.1	0.21
Self-reported diabetes (%)	4.6	5.9	3.5	5.9	2.6	4.5	0.53
10-year risk of first-onset CVD (%)	5.48 (5.28–5.67)	4.76 (4.40–5.11)	5.36 (4.97–5.75)	5.57 (5.18–5.95)	5.74 (5.27–6.20)	6.00 (5.42–6.58)	**0.002**
Average daily physical activity (cpm)	651 (637–664)	715 (681–749)	670 (642–699)	643 (616–670)	616 (588–645)	603 (572–634)	** < 0.001**
SED (%)	53.2 (52.5–53.9)	50.1 (48.5–51.8)	51.6 (50.1–53.1)	54.1 (52.9–55.3)	54.8 (53.3–56.3)	55.3 (53.6–57.1)	** < 0.001**
Average time in SED bout (min)	175 (168–181)	155 (140–169)	164 (151–177)	174 (163–185)	188 (173–202)	196 (180–213)	** < 0.001**
LIPA (%)	40.9 (40.3–41.5)	43.1 (41.6–44.6)	42.4 (41.0–43.8)	40.0 (38.9–41.1)	39.8 (38.4–41.1)	39.3 (37.8–40.9)	** < 0.001**
MVPA (%)	5.9 (5.7–6.1)	6.8 (6.2–7.3)	6.0 (5.6–6.4)	5.9 (5.4–6.4)	5.5 (5.0–5.9)	5.3 (4.8–5.8)	**0.001**
Average time in MVPA bout (min)	19 (18–20)	23 (19–26)	19 (16–21)	20 (17–23)	17 (15–20)	15 (12–17)	**0.002**
SED in the morning (%)	49.0 (48.1–49.9)	44.8 (42.5–47.0)	46.5 (44.6–48.3)	50.2 (48.6–51.8)	51.8 (49.7–53.8)	52.0 (49.6–54.4)	** < 0.001**
LIPA in the morning (%)	44.4 (43.6–45.2)	47.7 (45.6–49.7)	46.5 (44.9–48.2)	43.4 (41.9–44.9)	42.3 (40.5–44.1)	41.8 (39.7–44.0)	** < 0.001**
MVPA in the morning (%)	6.6 (6.3–6.9)	7.5 (6.8–8.3)	7.0 (6.4–7.6)	6.4 (5.9–7.0)	5.9 (5.2–6.6)	6.1 (5.4–6.9)	**0.007**
SED in the afternoon (%)	47.9 (47.1–48.6)	45.3 (43.5–47.0)	46.6 (44.8–48.4)	49.0 (47.6–50.5)	49.1 (47.5–50.7)	49.2 (47.2–51.1)	**0.002**
LIPA in the afternoon (%)	45.0 (44.3–45.7)	46.8 (45.2–48.4)	46.3 (44.7–48.0)	43.8 (42.5–45.2)	44.2 (42.7–45.8)	44.1 (42.3–46.0)	**0.018**
MVPA in the afternoon (%)	7.1 (6.8–7.4)	8.0 (7.3–8.7)	7.1 (6.5–7.6)	7.1 (6.5–7.7)	6.7 (6.0–7.3)	6.7 (6.1–7.3)	**0.036**
SED in the evening (%)	60.3 (59.5–61.0)	59.5 (57.8–61.2)	60.5 (58.7–62.3)	61.2 (59.7–62.7)	59.9 (58.2–61.6)	59.8 (58.0–61.7)	0.62
LIPA in the evening (%)	35.4 (34.8–36.1)	35.9 (34.5–37.4)	35.1 (33.6–36.7)	34.5 (33.3–35.7)	35.8 (34.3–37.3)	36.2 (34.6–37.8)	0.44
MVPA in the evening (%)	4.2 (3.9–4.5)	4.6 (4.0–5.2)	3.8 (3.4–4.2)	4.3 (3.6–5.0)	4.4 (3.8–5.0)	4.0 (3.4–4.5)	0.39

Data shown as percentage or mean (95% CI).

*cpm* counts per minute, *LIPA* light physical activity, *MVPA* moderate to vigorous intensity physical activity, *SED* time spent sedentary.

### Chronotype and mid-sleep time

**Figure 2:**
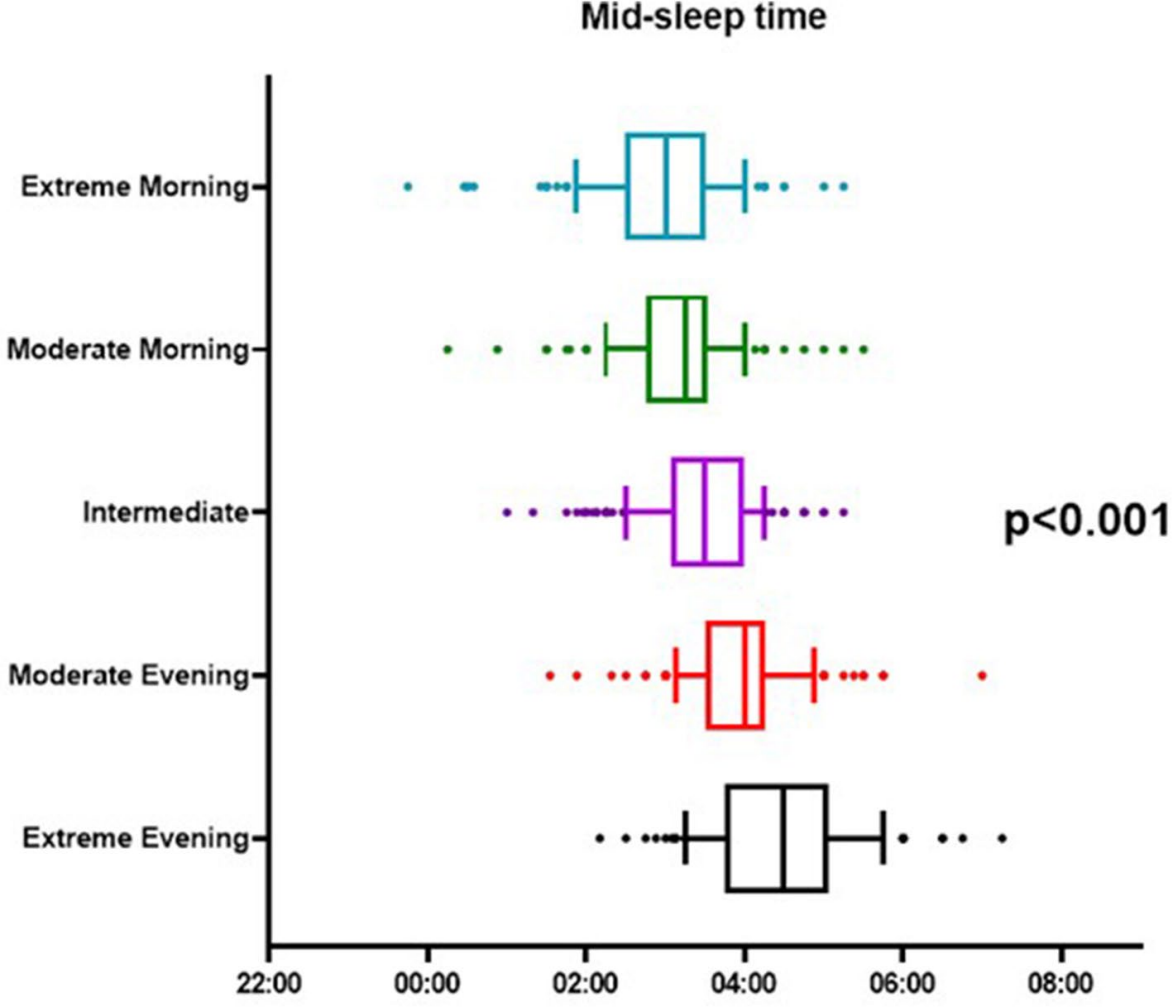
Mid-sleep time by chronotype (n = 685). The mid-sleep times of the 5 chronotypes from extreme morning to extreme evening were 2:55am, 3:11am, 3:29am, 3:58am, and 4:29am, respectively. There is a dose-dependent relationship between mid-sleep time and subjective chronotype (ANOVA, p < 0.001).

Mid-sleep time was compared among the chronotypes (n = 685), and there was a dose–response relationship between the two measures (p < 0.001, Fig. 2). Extreme morning chronotypes had the earliest average mid-sleep time, at 2:55 am, while extreme evening chronotypes had the latest average mid-sleep time at 4:29 am.

### Chronotype and physical activity

**Figure 3:**
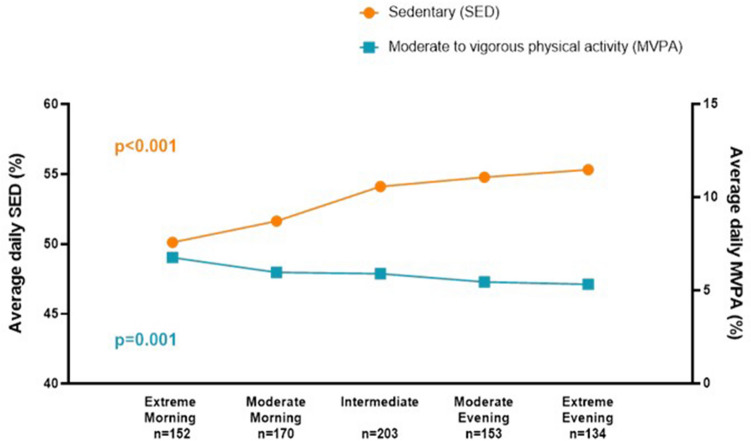
Physical activity pattern by chronotype. SED increases from extreme morning to extreme evening type (ANOVA, p < 0.001). MVPA decreases from extreme morning to extreme evening type (ANOVA, p = 0.001).

Physical activity patterns derived from accelerometry varied significantly across the chronotypes and is summarized in Table 1. Total activity counts were greatest among the extreme morning types and decreased in a dose-dependent manner throughout the chronotypes towards extreme evening type (p < 0.001). A similar relationship between chronotype and amount of physical activity is observed when analyzing MVPA and SED separately, with extreme morning types exhibiting more MVPA, while extreme evening types exhibit more SED (p = 0.001 and < 0.001, respectively) (Fig. 3).

Time spent in bouts of MVPA and SED also varied across chronotypes in a similar way to total daily MVPA and SED. Time spent in MVPA bouts was greatest among extreme morning types, and lowest among extreme evening types followed by moderate evening types (p = 0.002). Time spent in SED bouts was greatest among extreme evening types and decreased in a dose-dependent manner with the lowest time spent in SED bouts among extreme morning types (p < 0.001).

Regarding time of day of MVPA and SED, there was a significant variation during the morning and afternoon, which in the case of SED was entirely dose-dependent, with the highest amount of SED among extreme evening types and the lowest amount of SED among extreme morning types (p < 0.001 morning, p = 0.002 afternoon, Table 1). There was no significant variation across chronotypes during the evening. There was a similar trend with regards to MVPA, with the highest levels among extreme morning types. However, the lowest levels of morning and afternoon MVPA were found among moderate evening types.

In the multivariate regression analysis, compared to extreme morning chronotype, intermediate chronotype, as well as moderate/extreme evening chronotype was significantly associated with increased SED, whereas all other chronotypes were associated with decreased MVPA. This was after controlling for gender, age, BMI, waist circumference, SES, smoking, university education, work status, depression symptoms, hypertension, and self-reported diabetes mellitus (Table 2).

**Table 2:** Association between chronotypes and physical activity pattern in generalized linear regression models.

	Time spent sedentary (%)			Moderate to vigorous physical activity (%)		
	β	SE	P value	β	SE	P value
Extreme morning	Reference			Reference		
Moderate morning	0.73	1.04	0.48	− 0.68	0.34	**0.045**
Intermediate	2.79	1.00	**0.005**	− 0.82	0.33	**0.012**
Moderate evening	3.74	1.07	** < 0.001**	− 1.30	0.35	** < 0.001**
Extreme evening	3.64	1.12	**0.001**	− 1.19	0.36	**0.001**

Controlled for gender, age, body mass index, waist circumference, SES, smoking, unhealthy drinking, university education, work status, depression symptoms, hypertension and self-reported diabetes mellitus.

### Chronotype, physical activity and SCORE2

SCORE2 was lowest among extreme morning types (4.76 ± 2.21%) and increased in a dose-dependent manner with the highest risk among extreme evening types (p = 0.002). A generalized linear regression analysis revealed a significantly increased CVD risk among extreme evening types compared to extreme morning types when controlling for cofounders (β = 0.45, SE = 0.21, p = 0.031). In addition, a 10% increase in MVPA was associated with a 0.8% reduction of CVD risk (p = 0.001, Table 3). There was, however, no significant association between SED and CVD risk in this model.

**Table 3:** Associations of chronotypes and physical activity pattern on 10-year risk of first-onset cardiovascular disease.

	10-year risk of first-onset cardiovascular disease		
	β	SE	P value
Extreme morning vs	Reference		
Moderate morning	0.03	0.20	0.87
Intermediate	0.02	0.19	0.92
Moderate evening	0.10	0.20	0.62
Extreme evening	0.45	0.21	**0.031**
Moderate to vigorous physical activity (%)	− 0.08	0.02	**0.001**
Time spent sedentary (%)	0.00	0.01	1.00

Controlled for gender, age, body mass index, waist circumference, SES, smoking, unhealthy drinking, university education, work status and depression symptoms.

**Figure 4:**
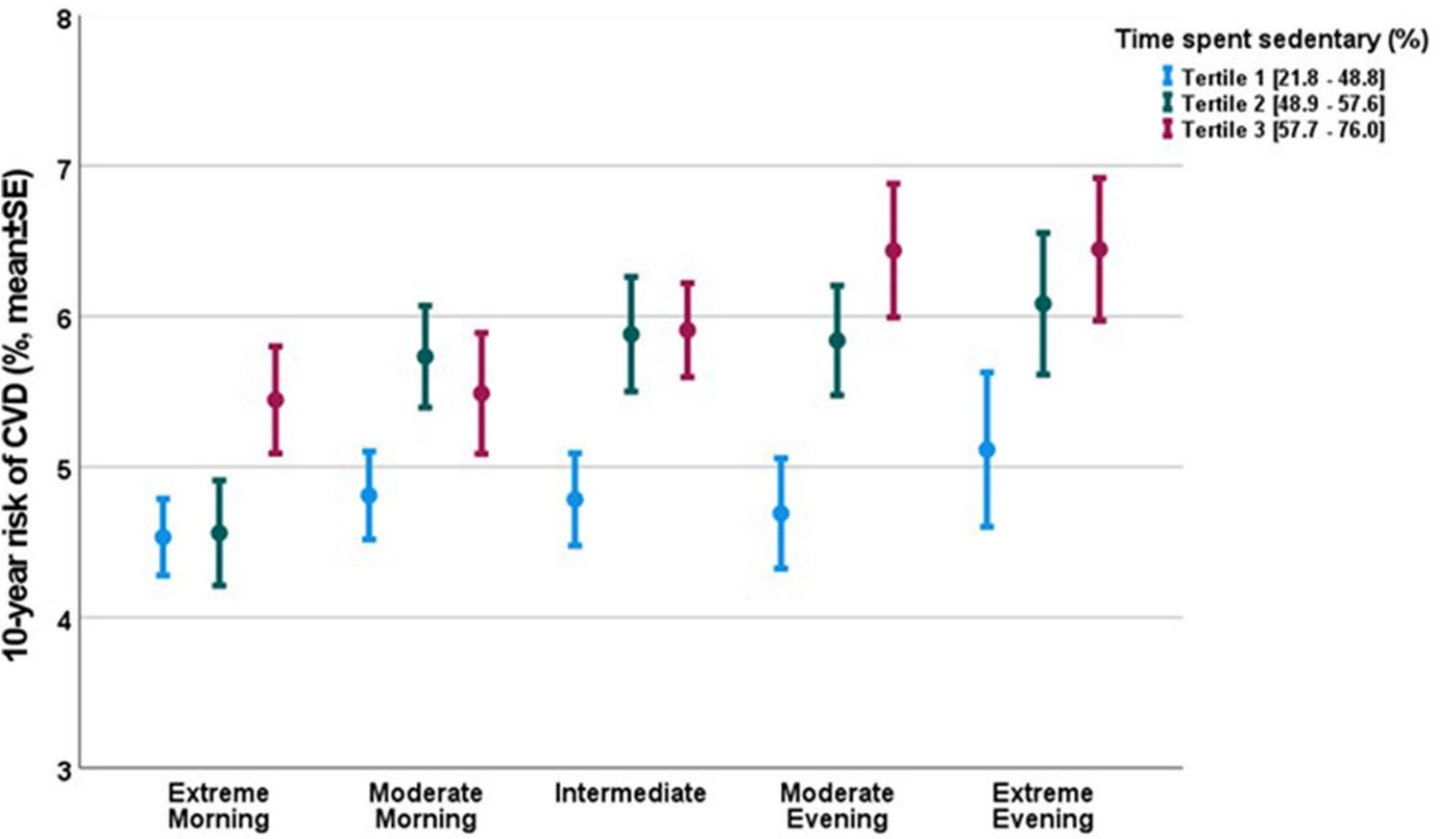
SCORE2 risk and time spent sedentary by chronotype. There is a dose-dependent relationship between chronotype and SCORE2 (ANOVA, p = 0.002). There is also a dose-dependent relationship between SED (represented in tertiles) and SCORE2 in the intermediate and moderate evening chronotypes (ANOVA, p = 0.034 and p = 0.013, respectively).

A sensitivity analysis excluding MVPA in the multivariate regression model confirmed the independent association between chronotype and CVD risk (extreme evening types vs. extreme morning types, β = 0.51, SE = 0.21, p = 0.016). There was also a statistical trend for an association between SED and CVD risk (β = 0.012, SE = 0.007, p = 0.076). The influence of SED on CVD risk was more pronounced among intermediate and moderate evening type subjects (p = 0.034 and 0.013, respectively) (Fig. 4).

Mediation analyses were performed in order to illustrate a possible mediation effect of physical activity pattern on the link between chronotype and CVD risk (Figure S1). The mediated effects of chronotype on SCORE2 through SED and MVPA were investigated using bootstrapping methods, and a significant mediation pathway via SED, but not MVPA was demonstrated (Table S2). The indirect effect via SED was approximately 10% when controlling for BMI, SES, depression symptoms, and MVPA (Table S3 & S4). Compared to extreme morning chronotypes, moderate morning types did not have increased CVD risk in this model. Interestingly, intermediate types did have an increased CVD risk, but this was mainly due to the mediating effect of SED, with a statistical trend to a direct impact on SCORE2. Moderate and extreme evening types were associated with a significantly increased SCORE2 due to both a direct link, and a link mediated by SED (Table S4).

## Discussion

**Figure 5:**
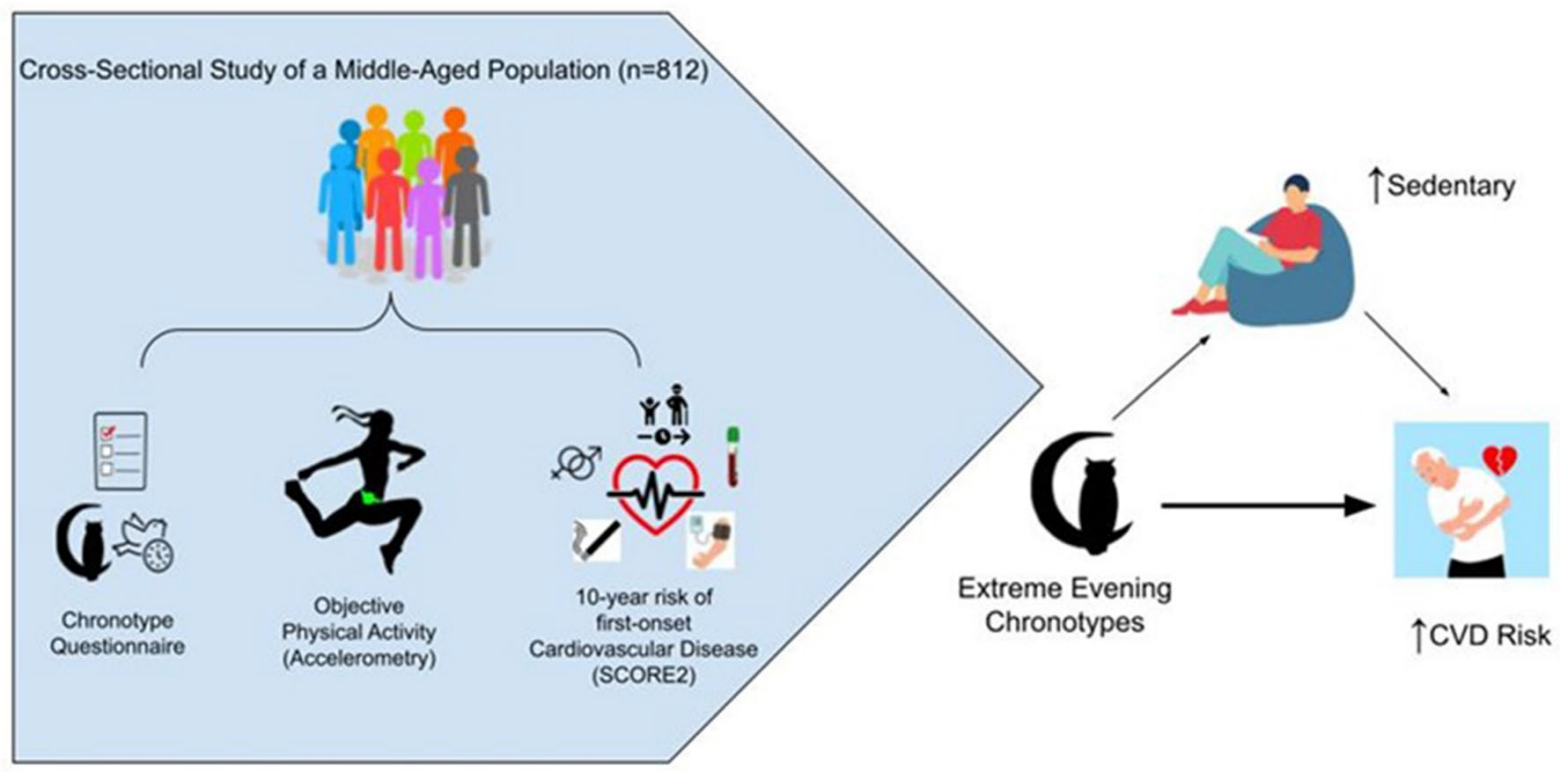
Study summary. Cross-sectional analysis of chronotype, physical activity by accelerometry, and 10-year risk of CVD was performed in a middle-aged cohort. Extreme evening chronotype was associated with increased 10-year risk of CVD compared to extreme morning types. This association was partially mediated by increased time spent sedentary among extreme evening types.

This is the first study in a large population-based cohort to establish an association between chronotype, objectively assessed physical activity pattern, and the 10-year risk of first-onset CVD as estimated by SCORE2. Using accelerometry data, we have shown that evening types are less active and more sedentary than morning types. This is in line with previous studies examining this relationship in other populations utilizing subjective and objective physical activity assessment^10–13^. Furthermore, we have also found that it is physical activity behavior in the morning and afternoon rather than during the evening which distinguishes morning types from evening types. SCORE2 is a state-of-the-art model for estimating the 10-year risk of first-onset CVD^21^. We have reported, for the first time, that extreme evening chronotype is independently associated with increased SCORE2 risk, supporting previous studies which suggest that evening chronotypes have poorer cardiovascular health than other chronotypes, with higher odds of arterial hypertension^33^, lower levels of HDL cholesterol^34^, and higher rates of CVD mortality ^20^. In addition, we have demonstrated that sedentary lifestyle is a possible mediator for part of the link between extreme evening chronotype and increased CVD risk. Figure 5 displays a summary of our methods and results. Our findings provide further insight on the interrelationship between the circadian timing system, lifestyle, and cardiometabolic function.

The circadian timing system, dictated by the cycle of day and night formed by earth’s rotation on its axis around the sun, is a fundamental aspect of human physiology and behavior. It orchestrates the temporal organization of virtually every biological process in our organism through a master molecular clock in the suprachiasmatic nuclei of the hypothalamus. This master clock exerts temporal control of endocrine and nervous function as well as molecular clocks found in peripheral tissues. As such, disruption in this system could set off a cascade of downstream effects on clock, endocrine and autonomic nervous function, resulting in altered physiological, behavioral and mental states^35^. For example, a number of studies in both humans and rodent models have indicated a link between molecular clock genes and blood pressure variation^36^. Other cardiometabolic factors such as heart rate, hormone secretion, glucose levels, vascular tone, coagulation, and inflammatory responses exhibit rhythmicity under circadian control^16^. Circadian disruption in humans by forced desynchrony and simulated shift work protocols have shown to have adverse metabolic and cardiovascular impacts such as increased blood pressure, decreased cardiac vagal modulation, decreased excretion rate of urinary adrenaline, and increased levels of inflammatory markers in serum^16,37^.

The human circadian timing system must constantly be resynchronized, or entrained, to the earth’s 24-h day because its endogenous period is slightly longer than 24 h. There are inter-individual differences both in endogenous period as well as in entrainment. Chronotype is a manifestation and marker of these variations, also influenced by psychological, behavioral, and environmental parameters which can alter the homeostatic drive for sleep and directly influence vigilance states^1,38^. Evening chronotype can thereby represent irregularities in the individual’s circadian timing system. These irregularities, apart from resulting in direct physiological disruptions, could also result in a desynchronization in relationship to the environment, be it the natural light–dark cycle, or socially imposed school and work schedules^39^.

There is no consensus on why evening types are less active and more likely to suffer from CVD than morning types, and it is likely multifactorial. Direct circadian disruption of physiology is presumably one main factor that increases the risk of CVD, as can be inferred from our analysis. Lifestyle, such as physical activity patterns are probably another major factor. We found that all chronotypes in our cohort had a similar level of physical activity during the evening, suggesting that evening types may be sedentary while morning types have already gone to bed. Factors such as societal norms and lack of daylight might discourage evening types from spending this time exercising. Sleep alterations, such as lower sleep quality and work-day sleep deprivation among evening types resulting in tiredness might also be an explanation for low physical activity and unhealthy behaviors^40^. This, however, is not evident in our cohort, as self-reported sleep duration and sleep quality did not differ between chronotypes. On the other hand, sleep timing did differ between chronotypes, possibly indicating that the later sleep timing in evening types is less restorative. This is supported by the fact that deep sleep occurs earlier in the night^41^. This difference in sleep timing might also signify the presence of social and/or environmental jetlag, resulting in circadian misalignment. Discrepancy between biological and environmental time can contribute to both psychological and physiological stress, which in turn could lead to suboptimal lifestyle and cardiovascular health^39,42^.

Our study is one of few to investigate the relationship between chronotype and objectively measured physical activity. This is meaningful, as subjective data are not entirely accurate since validity and reliability of physical activity questionnaires may be low^43^. This has also been demonstrated in our cohort^44^. Cardiovascular risk prediction is widely used in clinical practice to guide treatment decisions, and several matrixes have been applied^45^. SCORE2, a new model based on 13 million individuals and more than 60 000 incident CVD events during follow-up, has recently been proposed to predict the 10-year risk of first-onset CVD in European populations^21^. Both fatal and non-fatal cardiovascular events are considered to estimate total CVD burden. In addition, the model is recalibrated to four European regions with different CVD risk levels for better risk stratification. Our findings are therefore highly relevant in clinical preventative cardiology, as they provide a means of further fine-tuning clinical CVD risk assessment. These advances in the understanding of the relationship between CVD and circadian biology also support social change to for example adjust school start times^46^, regulate shift work^47^, and advocate for the abolishment of time change to improve health^48^.

Despite its wide scope and state-of-the art mapping, our study has some limitations. Firstly, circadian phase is most reliably measured by dim light melatonin onset which can also be reflected by sleep timing and subjectively assessed chronotype^1,49,50^. In the current study, we used a single question to determine chronotype which can be considered a proxy of circadian phase^20,49^. Importantly, we validated our chronotype measurements by comparing a subset with self-reported mid-sleep time, with cohesive responses to both questions^50^. There was an over 1.5-h difference in average mid-sleep time between extreme evening and morning chronotypes despite similar sleep duration. Secondly, similarly to other population-based studies of this kind, participants with poorer health and lower health-awareness turned down participation in SCAPIS to a greater extent^51^. Our study population was also in better health than the excluded subjects as individuals in poor health tended to avoid accelerometer recordings (Table S1). Thirdly, we had no information on light exposure, social jetlag and the four days of accelerometry did not distinguish between leisure versus work-related physical activity. Studies suggests that light exposure is an environmental and behavioral factor that can influence chronotype through manipulation of the circadian system, sleep homeostasis and a direct influence on physiology^52,53^. Fourthly, information on shift work schedules was lacking in the study and whether participants took this into account when evaluating their own chronotype was unknown. However, subjective chronotype was correlated with mid-sleep time on work-free days. Fifthly, the narrow age range of our cohort may limit the generalizability of the results. Finally, the cross-sectional design of the current study does not allow firm conclusions on causality regarding the relationship between chronotype and CVD risk.

The link we have established between chronotype, physical activity patterns, and CVD risk highlights the relevance of circadian factors in preventative cardiology and promotion of healthy lifestyle. Our data suggests that avoiding phase delay of the circadian timing system, a phenomenon which is increasingly experienced in modern society due to lack of dependence on natural environmental cues, are important^54^. Lifestyle promotion to encourage more physical activity and less sedentary behavior targeted especially towards evening types might also be an effective strategy to improve cardiovascular health in the population. For instance, the Swedish model of physical activity on prescription, which has been shown to increase the level of physical activity in insufficiently active patients includes an individualized dialogue, during which chronotype can be discussed^55^. An evidence gap has recently been highlighted concerning the inclusion of sleep disorders in cardiovascular risk prediction, as well as in the understanding of the pathways by which sleep disturbances are linked with CVD^56^. Future longitudinal and interventional studies are therefore needed to assess a potential causal relationship between chronotype, lifestyle factors and cardiometabolic morbidities. Prospective studies are also needed to establish efficacy of potential treatment strategies such as enforcement of the circadian system and phase advancement with light therapy or adjustment of school and work schedules to better suit the biology of “night owls”. We argue that consideration of chronotype, a potentially modifiable risk factor, is critical when promoting lifestyle interventions and developing individually tailored treatments to minimize the risk of CVD.

## Supplementary Information

Supplementary Information.
