# Supplementary material for: Eveningness is associated with sedentary behavior and increased 10-year risk of cardiovascular disease: the SCAPIS pilot cohort
Source: Sci Rep. 2022 May 17;12:8203. doi: 10.1038/s41598-022-12267-5 (PMC9113987; doi:10.1038/s41598-022-12267-5)

**Eveningness is associated with sedentary behavior and**

**increased 10-year risk of cardiovascular disease – the SCAPIS pilot cohort**

Mio Kobayashi Frisk^1^; Jan Hedner^1^; Ludger Grote^1,2^; Örjan Ekblom^3^; Daniel Arvidsson^4^; Göran Bergström^5,6^; Mats Börjesson^4,5,7^†; Ding Zou^1^†*

^1^ Center for Sleep and Vigilance Disorders, Department of Internal Medicine and Clinical Nutrition, Institute of Medicine, Sahlgrenska Academy, University of Gothenburg, Gothenburg, Sweden

^2^ Sleep Disorders Center, Department of Pulmonary Medicine, Sahlgrenska University Hospital, Gothenburg, Sweden

^3^ Department of Physical activity and Health, The Swedish School of Sport and Health Sciences, Stockholm, Sweden

^4^ Center for Health and Performance, Institute of Food, Nutrition and Sports Science, Gothenburg University, Gothenburg, Sweden

^5^ Department of Molecular and Clinical Medicine, Institute of Medicine, Sahlgrenska Academy, Gothenburg University, Gothenburg, Sweden

^6^ Department of Clinical Physiology, Region Västra Götaland, Sahlgrenska University Hospital, Gothenburg, Sweden

^7^ Sahlgrenska University Hospital/Östra, Gothenburg, Sweden

†Co-last authors

*Corresponding author

Running headline: Chronotype, sedentary behavior and CV risk

**Correspondence:**

Ding Zou, MD, PhD

Center for Sleep and Vigilance Disorders

Department of Internal Medicine and Clinical Nutrition

University of Gothenburg

Medicinaregatan 8b Box 421

SE-40530, Gothenburg

Sweden

E-mail: [zou.ding@lungall.gu.se](mailto:zou.ding@lungall.gu.se)

Telephone: +46 70 4383913

Fax: +46 31 825207

**Table S1.** Characteristics of included vs. excluded participants in the SCAPIS pilot cohort

|  | Included  (n=812) | Excluded  (n varies due to missing data) | p-value |
| --- | --- | --- | --- |
| Male gender (%) | 48.4 | 54.2 | 0.087 |
| Age (years) | 57.6 (57.3-57.9) | 57.8 (57.2-58.3) | 0.69 |
| Body mass index (BMI) (kg/m^2^) | 27.0 (26.7-27.3) | 28.1 (27.5-28.6) | **0.001** |
| Waist circumference (cm) | 94.6 (93.7-95.4) | 97.9 (96.3-99.5) | **<0.001** |
| Low socioeconomic status (%) | 45.1 | 62.2 | **<0.001** |
| University education (%) | 39.5 | 29.9 | **0.003** |
| Current/occasional/former smoker (%) | 55.4 | 61.1 | 0.093 |
| Unhealthy alcohol consumption (%) | 28.0 | 28.7 | 0.82 |
| Income-related job (%) | 78.9 | 61.9 | **<0.001** |
| Depression symptoms (%) | 25.6 | 32.0 | **0.044** |
| 10-year risk of first-onset cardiovascular disease (SCORE2) (%) | 5.48 (5.28-5.67) | 6.15 (5.80-6.50) | **0.001** |

**Table S2.** Summary of mediation analysis of time spent sedentary (SED) and moderate to vigorous intensity physical activity (MVPA) adjusted for BMI, socioeconomic status (SES) and depression symptoms

|  | Standardized coefficients | t | P value | Bootstrapping  [95%CI] |
| --- | --- | --- | --- | --- |
| **Model a1** |  |  |  |  |
| Chronotype 🡪 SED |  |  |  |  |
| Extreme morning vs. Moderate morning | 0.103 | 0.956 | 0.339 |  |
| Intermediate | 0.356 | 3.429 | **<0.001** |  |
| Moderate evening | 0.460 | 4.165 | **<0.001** |  |
| Extreme evening | 0.456 | 3.962 | **<0.001** |  |
| **Model a2** |  |  |  |  |
| Chronotype 🡪 MVPA |  |  |  |  |
| Extreme morning vs. Moderate morning | -0.240 | -2.180 | **0.030** |  |
| Intermediate | -0.265 | -2.511 | **0.012** |  |
| Moderate evening | -0.418 | -3.721 | **<0.001** |  |
| Extreme evening | -0.391 | -3.333 | **<0.001** |  |
| **Model b1** |  |  |  |  |
| SED 🡪 SCORE2 | 0.125 | 3.189 | **0.002** |  |
| **Model b2** |  |  |  |  |
| MVPA 🡪 SCORE2 | -0.045 | -1.171 | 0.242 |  |
| **Total effect model on SCORE2** |  |  |  |  |
| Extreme morning vs. Moderate morning | 0.210 | 1.911 | 0.056 |  |
| Intermediate | 0.257 | 2.439 | **0.015** |  |
| Moderate evening | 0.352 | 3.143 | **0.002** |  |
| Extreme evening | 0.413 | 3.535 | **<0.001** |  |
|  | Standardized direct effect |  |  |  |
| **Direct effect of chronotype 🡪 SCORE2** |  |  |  |  |
| Extreme morning vs. Moderate morning | 0.186 | 1.707 | 0.088 |  |
| Intermediate | 0.201 | 1.907 | 0.057 |  |
| Moderate evening | 0.276 | 2.454 | **0.014** |  |
| Extreme evening | 0.339 | 2.892 | **0.004** |  |
|  | Standardized indirect effect |  |  |  |
| **Indirect effect chronotype 🡪SED 🡪 SCORE2** |  |  |  |  |
| Extreme morning vs. Moderate morning | 0.013 |  |  | [-0.015 - 0.045] |
| Intermediate | 0.044 |  |  | **[0.014 - 0.085]** |
| Moderate evening | 0.058 |  |  | **[0.020 - 0.109]** |
| Extreme evening | 0.057 |  |  | **[0.019 - 0.107]** |
| **Indirect effect chronotype 🡪MVPA 🡪 SCORE2** |  |  |  |  |
| Extreme morning vs. Moderate morning | 0.011 |  |  | [-0.009 - 0.039] |
| Intermediate | 0.012 |  |  | [-0.008 - 0.044] |
| Moderate evening | 0.019 |  |  | [-0.014 - 0.060] |
| Extreme evening | 0.018 |  |  | [-0.013 - 0.057] |

Significant results marked in bold. Model suggested SED but not MVPA was a significant mediator of the relationship between chronotype and SCORE2.

**Table S3.** Summary of mediation analysis of SED (unadjusted)

|  | Standardized coefficients | t | P value | Bootstrapping  [95%CI] |
| --- | --- | --- | --- | --- |
| **Model a** |  |  |  |  |
| Extreme morning vs. Moderate morning | 0.154 | 1.404 | 0.161 |  |
| Intermediate | 0.405 | 3.844 | **<0.001** |  |
| Moderate evening | 0.474 | 4.206 | **<0.001** |  |
| Extreme evening | 0.528 | 4.535 | **<0.001** |  |
| **Model b** |  |  |  |  |
| Time spent sedentary | 0.162 | 4.629 | **<0.001** |  |
| **Total effect model** |  |  |  |  |
| Extreme morning vs. Moderate morning | 0.214 | 1.936 | 0.053 |  |
| Intermediate | 0.288 | 2.710 | **0.007** |  |
| Moderate evening | 0.350 | 3.077 | **0.002** |  |
| Extreme evening | 0.443 | 3.766 | **<0.001** |  |
|  | Standardized direct effect |  |  |  |
| **Direct effect of X on Y** |  |  |  |  |
| Extreme morning vs. Moderate morning | 0.189 | 1.730 | 0.084 |  |
| Intermediate | 0.223 | 2.098 | **0.036** |  |
| Moderate evening | 0.273 | 2.404 | **0.016** |  |
| Extreme evening | 0.357 | 3.036 | **0.003** |  |
|  | Standardized indirect effect |  |  |  |
| **Indirect effect of X on Y** |  |  |  |  |
| Extreme morning vs. Moderate morning | 0.025 |  |  | [-0.012 - 0.067] |
| Intermediate | 0.066 |  |  | **[0.027 - 0.113]** |
| Moderate evening | 0.077 |  |  | **[0.033 - 0.132]** |
| Extreme evening | 0.086 |  |  | **[0.038 - 0.145]** |

Significant results marked in bold. X=chronotype, Y=SCORE2, M=SED. Model a represents the association between X and M. Model b represents the association between M and Y (see figure S1). 19% of the relationship between increased SCORE2 among extreme evening types compared to extreme morning types was mediated by SED.

**Table S4.** Summary of mediation analysis of SED adjusted for BMI, SES, depression symptoms and MVPA

|  | Standardized coefficients | t | P value | Bootstrapping  [95%CI] |
| --- | --- | --- | --- | --- |
| **Model a** |  |  |  |  |
| Extreme morning vs. Moderate morning | 0.003 | 0.032 | 0.974 |  |
| Intermediate | 0.245 | 2.597 | **0.010** |  |
| Moderate evening | 0.285 | 2.828 | **0.005** |  |
| Extreme evening | 0.293 | 2.791 | **0.005** |  |
| **Model b** |  |  |  |  |
| Time spent sedentary | 0.125 | 3.189 | **0.002** |  |
| **Total effect model** |  |  |  |  |
| Extreme morning vs. Moderate morning | 0.187 | 1.701 | 0.089 |  |
| Intermediate | 0.231 | 2.195 | **0.028** |  |
| Moderate evening | 0.312 | 2.768 | **0.006** |  |
| Extreme evening | 0.375 | 3.202 | **0.001** |  |
|  | Standardized direct effect |  |  |  |
| **Direct effect of X on Y** |  |  |  |  |
| Extreme morning vs. Moderate morning | 0.186 | 1.707 | 0.088 |  |
| Intermediate | 0.201 | 1.907 | 0.057 |  |
| Moderate evening | 0.276 | 2.454 | **0.014** |  |
| Extreme evening | 0.339 | 2.892 | **0.004** |  |
|  | Standardized indirect effect |  |  |  |
| **Indirect effect of X on Y** |  |  |  |  |
| Extreme morning vs. Moderate morning | 0.0004 |  |  | [-0.027 - 0.026] |
| Intermediate | 0.031 |  |  | **[0.005 - 0.062]** |
| Moderate evening | 0.036 |  |  | **[0.008 - 0.071]** |
| Extreme evening | 0.037 |  |  | **[0.008 - 0.075]** |

Significant results marked in bold. X=chronotype, Y=SCORE2, M=SED. Model a represents the association between X and M. Model b represents the association between M and Y (see figure S1). 10% of the relationship between increased SCORE2 among extreme evening types compared to extreme morning types was mediated by SED in an adjusted model.

**Figure S1.** Illustration of mediation analysis of chronotype, SED and cardiovascular disease (CVD) risk


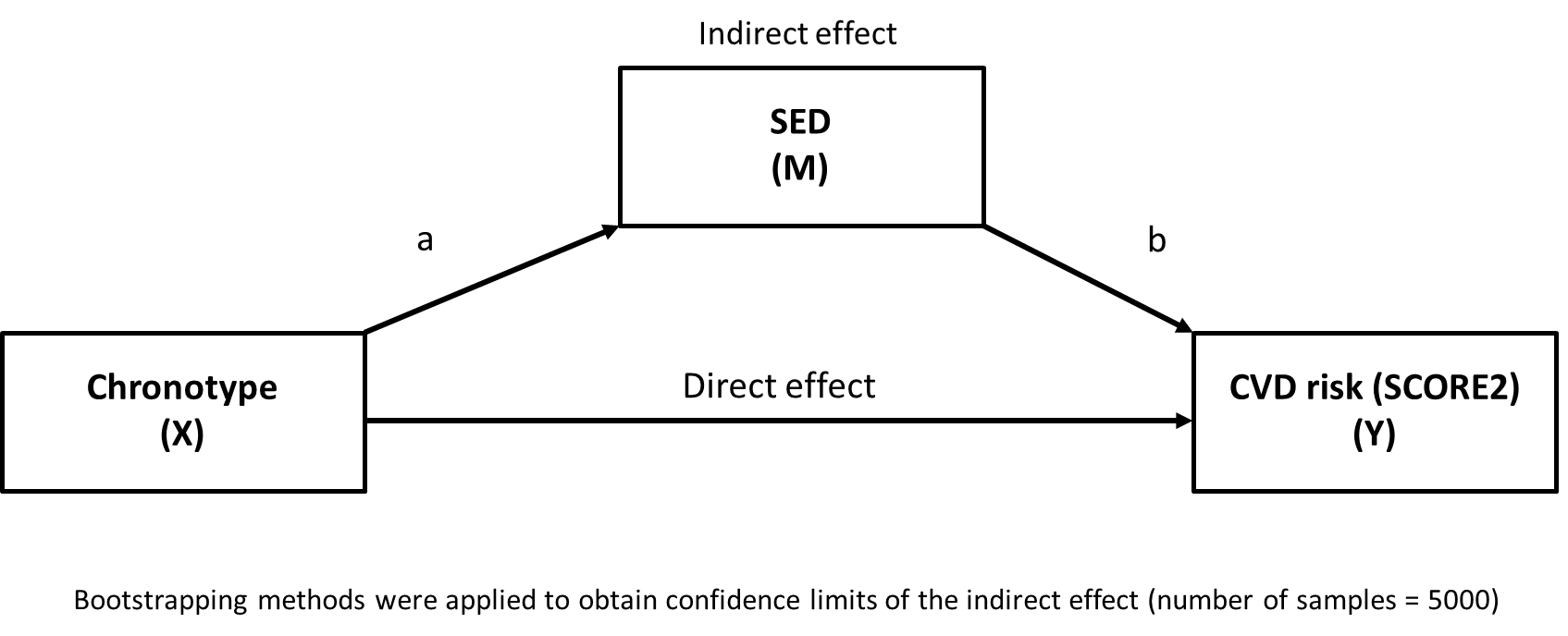

Supplement: Supplementary file 1 — Supplementary Information. [file 41598_2022_12267_MOESM1_ESM.docx]
